# Hydrogen Diffusion in Ti_3_C_2_ MXenes

**DOI:** 10.1021/acs.nanolett.4c05749

**Published:** 2025-02-14

**Authors:** Norbert H. Nickel

**Affiliations:** Helmholtz-Zentrum Berlin für Materialien und Energie, Nanoscale Solid−Liquid Interfaces, Schwarzschildstr. 8, 12489 Berlin, Germany

**Keywords:** hydrogen diffusion, Ti_3_C_2_ MXenes, diffusion coefficients, first-principles calculations

## Abstract

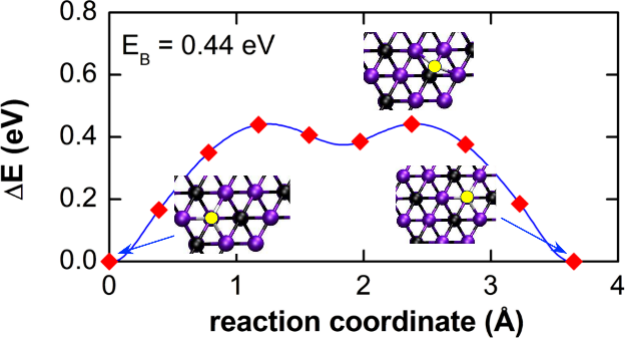

For energy storage in 2D MXenes using hydrogen, it is crucial to understand the relevant diffusion mechanisms. Density functional theory was used to determine hydrogen migration barriers, hopping frequencies, enthalpy and entropy of vacancy formation, and the diffusion coefficient of various possible migration paths. The results show that H diffusion is primarily governed by interstitial diffusion. For all investigated diffusion paths, the diffusion coefficients and prefactors, *D*_0_, the corresponding activation energy, *E*, and the hopping frequencies are calculated from the ab initio approach.

Two dimensional (2D) materials such as Ti_3_C_2_, which belong to the MXene group,^1^ have promising structural and electronic properties that render them highly interesting for energy harvesting and storage.^2−4^ The basic formula of MXenes is given as M_*n*+1_X_*n*_T_*y*_, where M denotes a transition metal, X is replaced by C or N atoms, and T represents the surface termination. The potential for energy storage of 2D MXenes is directly related to their large aspect ratio as well as their mechanical strength and flexibility.^5^ Based on molecular dynamics simulations, it has been suggested that 3.4 wt % of hydrogen can be adsorbed in and released from MXenes.^6^ A first experimental confirmation that hydrogen is soluble in Ti_3_C_2_ was obtained from neutron scattering experiments.^7^ At MXene surfaces, hydrogen dissociation can occur. This is corroborated by ab initio calculations.^8^ As a consequence and driven by the hydrogen chemical potential, H atoms and molecules will diffuse into the 2D MXene sheets.

In semiconductors and metals hydrogen is a ubiquitous diffusant causing both beneficial and detrimental effects even at low temperatures.^9−12^ Diffusion is driven by thermal energy and concentration gradients, facilitating the movement of atoms and ions within the material. Understanding these mechanisms is crucial for controlling the material properties. With regard to energy storage applications aimed at storing hydrogen, the microscopic diffusion processes of H atoms in MXenes are becoming increasingly important. In semiconductors and metals, common diffusion mechanisms include vacancy-mediated and interstitial diffusion. In the former case, the diffusing atom moves into an adjacent vacant site, thereby swapping its place with the vacancy. This process requires the presence of nearby vacancies and depends on the vacancy concentration. On the other hand, for interstitial diffusion hydrogen atoms move between lattice sites, and the only requirement for migration is an empty interstitial site.

In this paper, vacancy-mediated and interstitial diffusion of hydrogen in Ti_3_C_2_ is investigated using density functional theory. The temperature dependence of the diffusion coefficient is determined by calculating the H migration barriers, the jump frequencies, the enthalpies of vacancy formation, and their entropy of formation. It is shown that H diffusion in Ti_3_C_2_ is governed by interstitial diffusion, while vacancy-mediated diffusion hardly plays a role.

Interstitial and vacancy-mediated diffusion mechanisms are expected for hydrogen diffusion. The diffusion coefficient, *D*, is determined by microscopic parameters that include the hopping frequency and distance as well as the concentration of available empty sites. Since Ti_3_C_2_ MXenes crystallize in the hexagonal closed-packed structure, diffusion is anisotropic. Hence, the diffusion coefficient is given by^13^
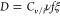
1where *C*_*v*/*i*_ is the vacancy or interstitial concentration, ν is the hopping frequency, and *f* is the diffusion correlation factor, which takes into account that atom jumps do not occur with equal probability in each direction.^14^ The factor *ξ* depends on the crystallographic direction of the diffusion process. In Ti_3_C_2_ MXenes hydrogen diffusion along the basal plane is limited to jumps in one atomic plane. Jumps in the *a*-direction but into an adjacent basal plane are highly unlikely. This would require the presence of Ti vacancies that are present in very low concentrations, since their enthalpy of formation amounts to 5.51–6.37 eV depending on the surface passivation of Ti_3_C_2_. For diffusion in the basal plane, the factor amounts to *ξ* = 3/2*a*^2^, where *a* is the lattice constant in the basal plane. On the other hand, for diffusion along the *c*-axis the factor changes to *ξ* = 3/4*c*^2^ where *c* is the corresponding lattice constant.

The vacancy concentration depends on temperature and is given by^13^
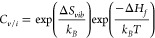
2where *k*_*B*_ is the Boltzmann constant, Δ*S*_*vib*_ describes the vibrational entropy due to vacancy formation, and Δ*H*_*f*_ is the enthalpy of vacancy formation, which is given by the difference of the total energies of the supercell with the vacancy and the ideal supercell and taking into account the chemical potential of the removed atom. On the other hand, interstitial sites are always present; hence *C*_*i*_ = 1.

The hopping frequencies can be obtained from transition state theory^15,16^ according to
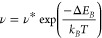
3

Here, Δ*E*_*B*_ is the migration barrier and the effective frequency is given by^15,16^
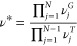
4

The products are formed by the frequencies of the initial equilibrium lattice position of the diffusing atom, ν_*j*_^*G*^, and the frequencies at the saddle point or transition state along the migration path, ν_*j*_^*T*^. At the transition state, frequencies corresponding to the unstable mode are excluded. Thus, the diffusion coefficient can be calculated from the lattice constants, formation energies of vacancies, saddle-point energies of the migration paths, associated effective frequencies, and vibrational entropy of vacancy formation.

A first-principles approach using density functional theory (DFT) was used to calculate the quantities required for the diffusion coefficient. For this purpose Ti_3_C_2_ layers with and without a surface passivation were modeled in a slap geometry. The supercells had rectangular and hexagonal shapes that consisted of up to 80 atoms for single layers and 109 atoms for double layers. The influence of surface termination on the diffusion coefficient was investigated by adding O–H groups to the MXene layer. The vacuum region above the surface extended to 10.34 Å. The calculations were performed with the Vienna Ab initio Simulation Package (VASP).^17,18^ The generalized gradient approximation (GGA) was used following the implementation of Perdew, Burke, and Ernzerhof.^19^ The Ti *s* and *d* electrons were considered as valence electrons in the projector-augmented wave method. Furthermore, the LDA+*U* approximation was used with an onsite Coulomb interaction of 2.8 eV for Ti.^20^ A plane wave cutoff of 700 eV and a *k*-point mesh of 3 × 3 × 1 were used. For the calculation of the migration barriers the nudged elastic band (NEB) method with the modified climbing-image technique was utilized.^21^ The vibrational frequencies of the H atom in the equilibrium and transition states were determined by calculating the vibrational modes. For this purpose, force constant calculations were performed by using the harmonic approximation. The vibrational entropy of vacancy formation was calculated with the program package phonopy^22,23^ using the harmonic approximation.

First, interstitial H diffusion is considered. In the case of interstitial diffusion, all possible hopping sites around the migrating atom are always unoccupied. Hence, to determine the diffusion coefficient, only the effective frequency and the migration barrier have to be calculated. In Figure 1 the diffusion barriers for surface and interstitial diffusion in the basal plane are shown as examples. At the surface of Ti_3_C_2_ the lowest energy site for a hydrogen atom is the hexagonal site with three Ti atoms as the nearest neighbors. Surface diffusion takes place between these low-energy sites. The change of the total energy, Δ*E*, as a function of the position of the hydrogen atom along the migration path is shown in Figure 1(a). Two maxima are observed that indicate a diffusion barrier of Δ*E*_*B*_ = 0.44 eV. These maxima arise when the H atom moves over the Ti–C bond, where the Ti atom is the next nearest neighbor and the adjacent C atom is in the second atomic layer.

**Figure 1:**
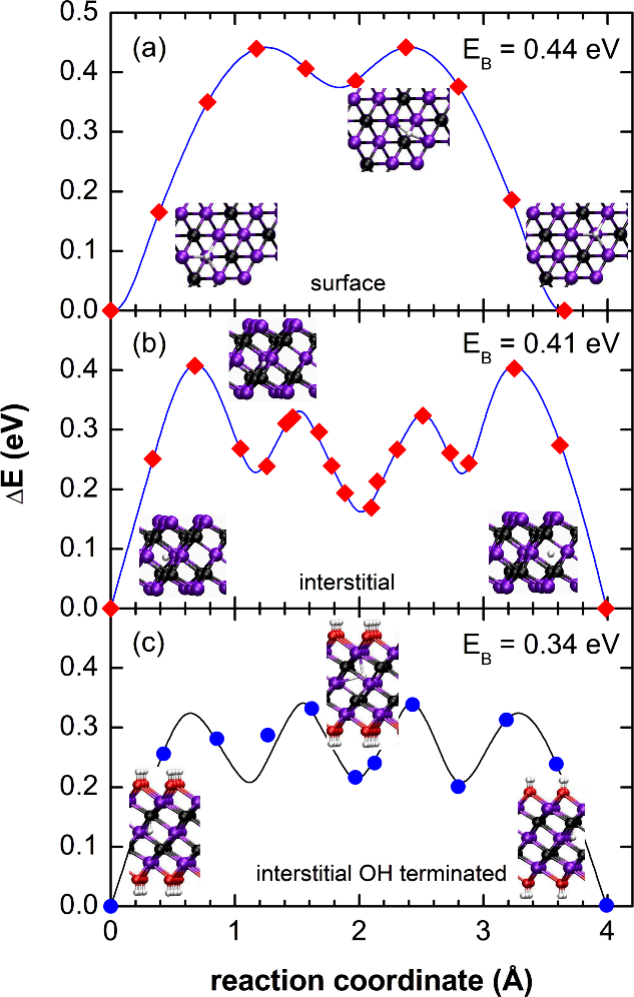
Change of the total energy, Δ*E*, for hydrogen diffusion on the surface of Ti_3_C_2_ (a) and along interstitial sites within an unpassivated (b) and O–H passivated MXene layer (c). The initial configurations, the transition states, and the final configurations are represented by the schematic crystal-structure images. Black, purple, red, and white spheres represent C, Ti, O, and H atoms, respectively. For surface diffusion the migration barrier amounts to *E*_*B*_ = 0.44 eV. In the case of interstitial H diffusion the energy barrier amounts to *E*_*B*_ = 0.41 and 0.34 eV, for unpassivated and O–H passivated Ti_3_C_2_, respectively.

Interstitial H inside a Ti_3_C_2_ sheet forms bonds to three nearest neighbor Ti atoms and to the nearest neighbor C atom.^8^ Thus, H diffusion requires breaking of these bonds. In Figure 1(b) the change of the total energy, Δ*E*, is shown as a function of the reaction coordinate for unpassivated Ti_3_C_2_. The barrier to overcome amounts to Δ*E*_*B*_ = 0.41 eV and is located at reaction coordinates of 0.68 and 3.25 Å. At both positions the H atom is located between two Ti atoms of the center layer but shifted slightly toward the adjacent C atom (see crystal structure images in Figure 1(b)). The two smaller maxima located at a reaction coordinate of ≈1.5 and ≈2.5 Å occur because the H atom moves through the high charge density of the interstitial lattice. Surmounting these barriers requires a relative energy of 0.1 and 0.16 eV.

For 2D materials, the migration barrier can depend on the surface termination. Therefore, the energy barrier for interstitial H diffusion in O–H terminated MXene was calculated. As both outer maxima become smaller, the diffusion barrier shifts along the reaction coordinate to the middle maxima (Figure 1(c)). Compared with unpassivated Ti_3_C_2_, the migration barrier decreases to Δ*E*_*B*_ = 0.34 eV.

The migration barriers were also calculated for H diffusion between two sheets of Ti_3_C_2_. In this case, H can migrate along the *c*-axis and in the basal plane. For these migration paths migration barriers of Δ*E*_*B*_ = 0.61 and 0.32 eV are obtained for diffusion along the basal plane and *c*-axis, respectively.

The second important diffusion mechanism is vacancy-mediated diffusion. However, for this migration process, the calculated diffusion barriers are considerably higher compared to interstitial H migration. In the basal plane and along the *c*-axis the diffusion barriers amount to Δ*E*_*B*_ = 0.52 and 0.92 eV, respectively, for unpassivated Ti_3_C_2_ (see Figure 2). An O–H surface passivation of the MXene layer results in an increase of the migration barrier for vacancy-mediated H diffusion in the basal plane to Δ*E*_*B*_ = 1.1 eV. The migration barriers for all investigated diffusion paths are summarized in Table 1, and schematic depictions of the diffusion paths are shown in Figure S1 in the Supporting Information.

**Figure 2:**
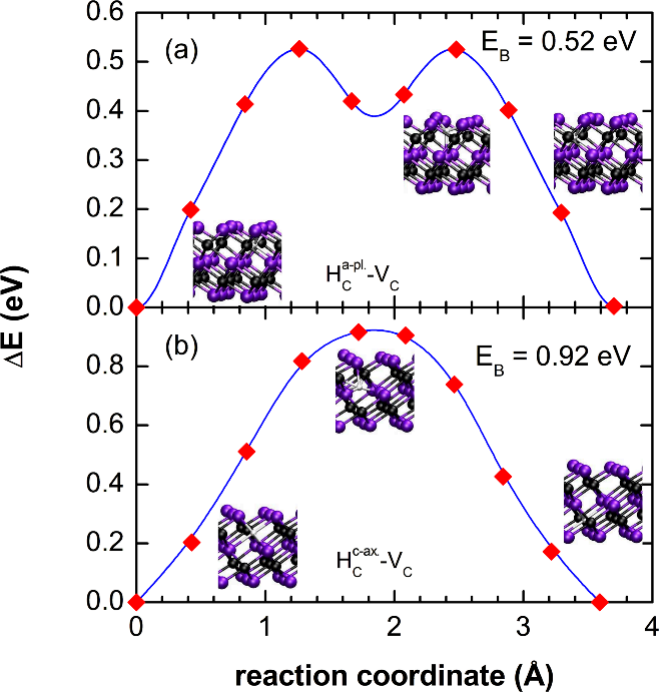
Change of the total energy, Δ*E*, for hydrogen-vacancy diffusion in the basal plane (a) and along the *c*-axis (b) of Ti_3_C_2_. The initial configurations, the transition states, and the final configurations are represented by the schematic crystal-structure images. The values of the diffusion barriers are shown in the figure.

**Table 1:** Calculated Energies of the Effective Jump Frequency, ν*, the Migration Barrier, Δ*E*_*B*_, and the Diffusion Activation Energy *E* = Δ*E*_*B*_ + Δ*H*_*f*_, for Various Interstitial and Vacancy-Mediated Diffusion Paths of Monatomic and Molecular Hydrogen in Ti_3_C_2_

diffusion path	ν* (THz)	Δ*E*_*B*_ (eV)	*E* (eV)
H_*i*_^*surf*^	21.1	0.44	
H_*i*_^*bulk*^	19.9	0.41	
H_*i*_^*bulk*^:OH	46.0	0.34	
H_*i*_^*c*-*ax*.,*btw*.^	39.8	0.32	
H_*i*_^*a*-*pl*.,*btw*.^	25.2	0.61	
H_*c*_^*c*-*ax*.^-V_*c*_	5.3	0.92	2.97
H_*c*_^*a*-*pl*.^-V_*c*_	6.8	0.52	2.57
H_*c*_^*a*-*pl*.^-V_*c*_:OH	6.9	1.10	2.47
H _2,*i*_^*surf*^	12.5	0.42	
H _2,*i*_^*bulk*^	18.1	0.32	

The effective jump frequencies, ν*, for each diffusion path vary between 5.3 and 46.0 THz (see Table 1). In combination with the H migration barrier, the temperature dependence of the hopping frequencies is obtained from eq 3. The temperature dependence of ν is shown in Figure 3. The magnitude of the hopping frequency shows a pronounced dependence on the effective jump frequency, ν*, and the associated migration barrier, *E*_*B*_. The highest hopping frequencies are obtained for interstitial diffusion and diffusion between two MXene sheets. For molecular H the hopping frequencies at the surface and in the bulk of Ti_3_C_2_ are comparable to those for monatomic H (see dash-dotted curves in Figure 3). On the other hand, the lowest hopping frequencies are obtained for vacancy-mediated H migration along the basal plane in OH-terminated Ti_3_C_2_ (dotted curve in Figure 2). This is mainly due to the large migration barrier of Δ*E*_*B*_ = 1.1 eV for this diffusion path.

**Figure 3:**
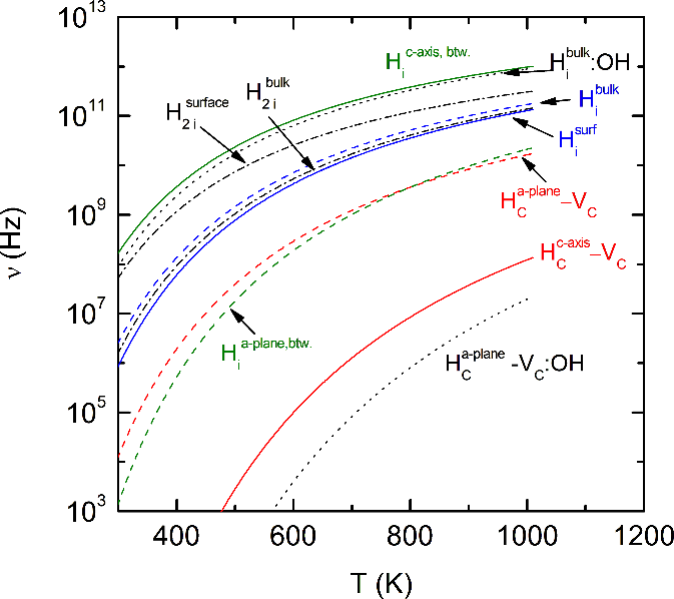
Hopping frequency as a function of temperature for interstitial and vacancy-mediated diffusion of H atoms and molecules in unpassivated and O–H terminated Ti_3_C_2_ MXene.

To calculate the diffusion coefficients, two additional quantities must be determined. The migrating H atom can exchange its place more than once with a vacant site. This is described by the correlation factor. For interstitial diffusion, successive jumps of an atom are strictly random and do not depend on the movement of an other atom. Hence, the correlation factor is equal to one.^24^ On the other hand, for vacancy-mediated diffusion in a solid with hexagonal close packing Compaan and Haven obtained a correlation factor of *f* = 0.781.^25^

The concentration of vacancies plays a key role in vacancy-mediated diffusion. It depends on the formation enthalpy and the vibrational entropy. In stoichiometric unpassivated Ti_3_C_2_ the formation enthalpies for Ti and C vacancies amount to 6.37 and 2.05 eV, respectively. If the surfaces are passivated with OH groups, the formation enthalpies decrease to 5.51 and 1.37 eV for the Ti and C vacancies, respectively. Because of the large formation enthalpies, the concentration of Ti vacancies will be much lower than the concentration of C vacancies. Consequently, a measurable contribution to hydrogen transport would occur only via C vacancies. The second term that affects the vacancy concentration is the vibrational entropy of the vacancy formation. For the formation of C vacancies a value of Δ*S*_*vib*_ = 1.804*k*_*B*_ was obtained.

Combining migration barriers, vacancy formation enthalpies, vibrational entropy, and the hopping frequencies, the diffusion coefficients were calculated for the interstitial and vacancy-mediated H migration paths according to eq 1. The temperature dependence of *D* is shown in Figure 4. The data clearly show that the dominant diffusion mechanism for H atoms is diffusion along the interstitial lattice sites. Even at elevated temperatures the diffusion coefficient for interstitial diffusion is more than 10 orders of magnitude larger than *D* for vacancy-mediated diffusion. The highest diffusion coefficients are obtained for interstitial H diffusion in the bulk of O–H terminated MXene (H_*i*_^*bulk*^:OH) and interstitial H diffusion between two Ti_3_C_2_ sheets along the basal plane (H_*i*_^*a*-*pl*.,*btw*.^). H diffusion at the surface and in the bulk of unpassivated Ti_3_C_2_ is comparable. *D* differs only by about a factor of 1.4–2.5 in the investigated temperature range (see Figure 4). Molecular hydrogen also diffuses easily in the basal plane and at the surface. The diffusion coefficients are comparable to those obtained for monatomic H migration (dashed-dotted curves in Figure 4). For energy-relevant applications based on hydrogen storage it is important to note that hydrogen migrates easily at moderate temperatures.

**Figure 4:**
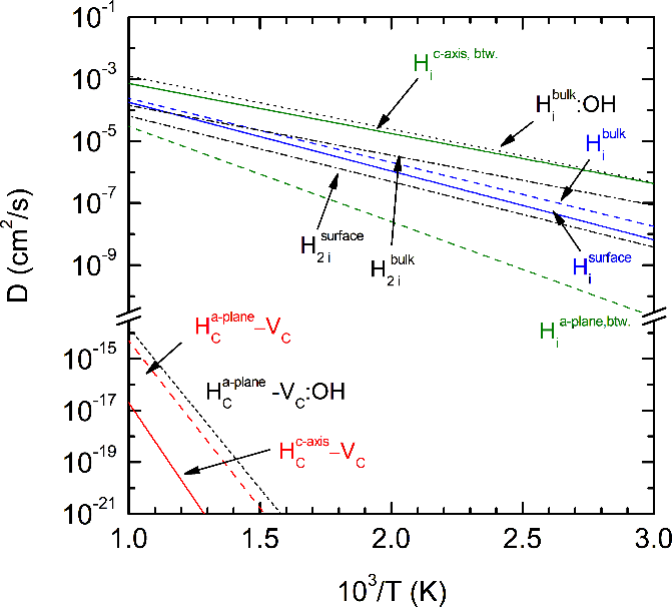
Diffusion coefficient vs temperature for interstitial and vacancy-mediated diffusion of H atoms and molecules in Ti_3_C_2_ MXene.

Vacancy-mediated diffusion owes its small diffusion coefficients mainly to the large enthalpy of formation of the C vacancies and thus their low concentration. Although the modification of the Ti_3_C_2_ surface by adding OH groups results in a decrease of the formation enthalpy for vacancies, and consequently a decrease of the activation energy, *E*, by 0.1 eV, the diffusion coefficient increases only by about a factor of 4 (see black dotted and red dashed curves in Figure 4). This makes vacancy-mediated H diffusion negligible.

From eqs 1–4 the diffusion prefactor is given by
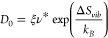
5and the activation energy can be written as *E* = Δ*E*_*B*_ + Δ*H*_*f*_. In Figure 4*D*_0_ is plotted as a function of the activation energy, *E*. Independent of the diffusion mechanism *D*_0_ varies between 5.9 × 10^–3^ and 6.5 × 10^–2^ cm^–2^/s.

In summary, for energy storage in 2D MXenes using hydrogen, it is important to understand the governing diffusion mechanisms. To establish the diffusion coefficients, all relevant microscopic parameters for the diffusion process were determined from ab initio calculations. This comprised the calculation of the hopping frequencies, hydrogen migration barriers, vacancy formation enthalpies, and entropy of vacancy formation. The results clearly show that hydrogen diffuses rapidly in Ti_3_C_2_. Moreover, H migration is governed by interstitial diffusion. The highest diffusion coefficients are obtained form O–H terminated MXene with values of 2.4 × 10^–5^ cm^–2^/s at a moderate temperature of 500 K. Based on the ab initio approach the diffusion prefactor, *D*_0_, and the activation energy, *E*, were determined for the investigated H diffusion paths. The diffusion prefactor varies between *D*_0_ = 5.9 × 10^–3^ and 6.5 × 10^–2^ cm^–2^/s in the energy range of *E* = 0–2.97 eV.

**Figure 5:**
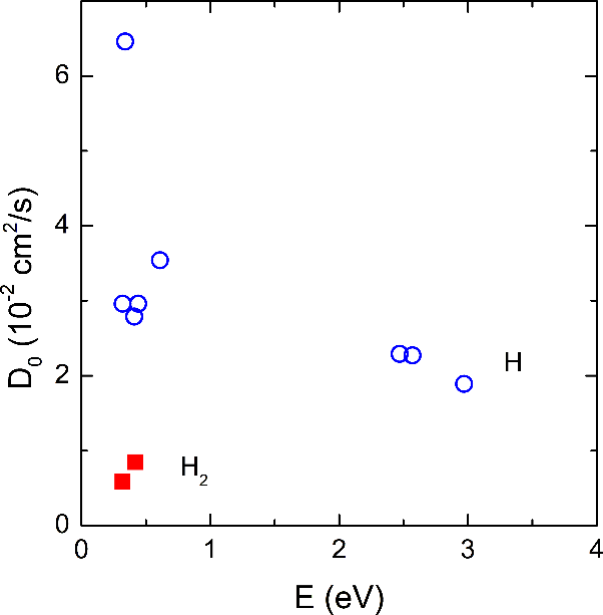
Diffusion prefactor, *D*_0_, as a function of the activation energy, *E* = Δ*E*_*B*_ + Δ*H*_*f*_, for monatomic and molecular H migration.

## Supporting Information Available

Schematic depiction of the diffusion paths (PDF)
