# Supplementary material for: Hydrogen Diffusion in Ti3C2 MXenes
Source: Nano Lett. 2025 Feb 14;25(8):3199–203. doi: 10.1021/acs.nanolett.4c05749 (PMC11869355; doi:10.1021/acs.nanolett.4c05749)
Supplement: Supplementary file 1 — nl4c05749_si_001.pdf [file nl4c05749_si_001.pdf]

Supporting Information for

Hydrogen diffusion in Ti_3C2 MXenes

Norbert. H. Nickel
Helmholtz-Zentrum Berlin für Materialien und Energie,
Nanoscale Solid-Liquid Interfaces, Schwarzschildstr. 8, 12489 Berlin, Germany

The investigated hydrogen migration paths for interstital and vacancy-mediated diffusion (see Tab. 1 of the paper) are schematically depicted in Fig. S1. For reasons of clarity, the OH surface passivation has been omitted from the illustration.

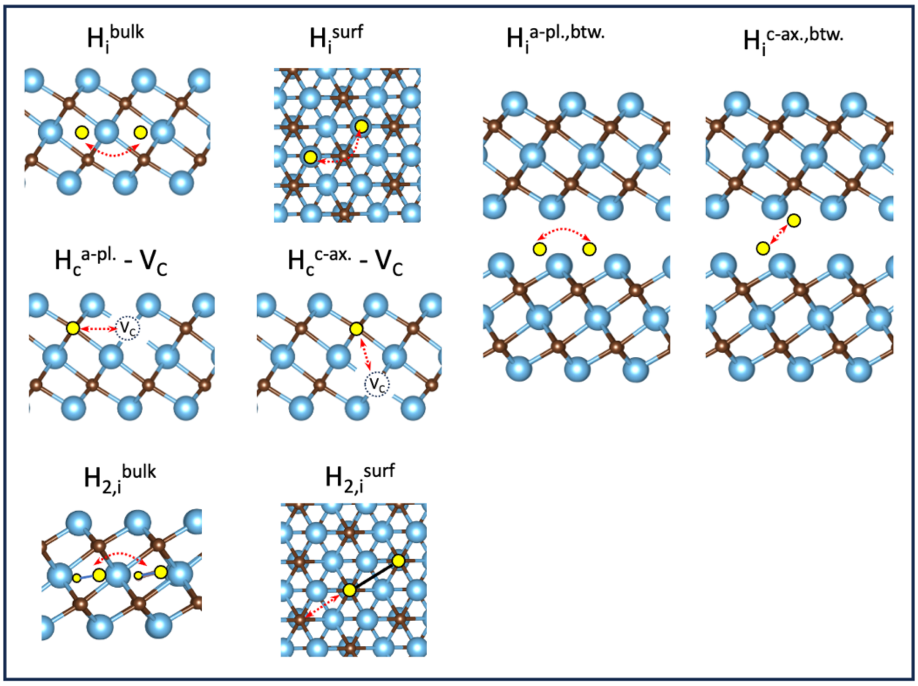

Fig. S1. Schematic depiction of the investigated diffusion paths for hydrogen migration in Ti_3C_2. The diffusion parameters obtained from ab-initio calculations are summarized in Tab.1 of the paper. Ti, C, and H atoms are depiced by blue, brown, and yellow circles. V_C denotes a carbon vacancy. The OH termination used for some of the caluclations is omitted for clarity.
